# Data on draft genome assembly and annotation of Haloxylon salicornicum Moq.

**DOI:** 10.1016/j.dib.2021.107721

**Published:** 2021-12-16

**Authors:** Fadila Al Salameen, Nazima Habibi, Sami Al Amad, Bashayer Al Doaij

**Affiliations:** Environment and Life Sciences Research Centre, Kuwait Institute for Scientific Research, Kuwait

**Keywords:** Whole-genome sequencing, Desert, Native plants, Biodiversity

## Abstract

*Haloxylon salicornicum* Moq. Bunge ex Boiss (Rimth) is one of the main structural elements in Eastern Arabian vegetation associations. The plant is utilized as a food source for domestic stock, stabilizes the soil surface besides providing suitable microclimates for exotic species. It is considered one of the most promising species for re-vegetation. *H. salicornicum* community is under threat from overgrazing leading to a reduction in the percentage of distribution from 22.7% to 2.2% in Kuwait. Therefore, genome characterization of this important Kuwaiti plant is required to formulate strategies for its conservation. Here we report the draft of the *H. salicornicum* genome, which was sequenced on an Illumina HiSeq 2500 platform. BUSCO assessment revealed 69% of the genome was to be complete. Overall, 12960 gene structures, 11280 protein-coding genes, 11309 mRNAs (protein-coding), 51265 exons and 48100 CDSs were predicted. Functional annotation was carried out by interproscan-5.29-68.0. A total of 7222 protein-coding sequences were, annotated out of 11309 by at least one ontology term. All these genes were associated with 11 major biological processes branched into 60 child processes.

## Specifications Table

**Table:** 

Subject	Plant Sciences
Specific subject area	Genomics
Type of data	Tables, Figures
How the data were acquired	Paired-end Illumina Sequencing
Data format	Raw, filtered, analysed
Parameters for data collection	A single specimen growing in its natural habitat (Al Kabd, Kuwait) was used for this study. Genomic DNA for sequencing was extracted from young leaves.
Description of data collection	Genomic DNA was digested using the restriction enzymes PstI+BtgI (New England BioLabs, Inc., Ipswich, MA, United States), and barcoded adapters were ligated to the DNA sample using T4 ligase (New England BioLabs, Inc.). Dual indexed libraries for *H. salicornicum* were pooled and loaded across 4 lanes of a 150 bp paired read sequencing run on an Illumina HiSeq 2500 (Illumina, San Diego, CA).
Data source location	Kuwait Institute for Scientific Research, Kuwait (N-DM-29.64798; E-DM-47.99595)
Data accessibility	Repository name: National Centre for Biotechnology Information Data identification number: PRJNA766761(SRA: SRR16094057) Direct URL to data: https://www.ncbi.nlm.nih.gov/sra/SRX12380181[accn] Supplementary data available at: https://figshare.com/s/a3c215093885a9707613

## Value of the Data

• The data provides valuable information on the genome sequences of *Haloxylon salicornicum* and fills in the gap of genomic studies in this genus.
• The genome assembly will be useful for geneticists interested in comparative genomics, conservation, breeding and phylogeny of *Haloxylon*.
• The genome analysis formulates a basis for further high depth sequencing of the species.
• The data can be used to develop molecular markers.

## 1 Data Description

The loss of biodiversity in arid lands due to harsh climatic conditions is an issue of global concern [1]. Human interventions and encroachment have further added to the effect. The native vegetation of Kuwait is unique with diverse species of desert plants adaptable to the harsh climate, however is degrading at an alarming rate. Native vegetation is crucial for the health of the environment, supporting agricultural productivity as well as the biodiversity that is central to a country's cultural identity. To formulate effective conservation and restoration strategies, advanced molecular research is highly desirable [2], [3], [4]. Genome sequencing studies are thus helpful in providing first-hand information on the genome size, repeat content, microsatellite regions and genes involved in local adaptation. Synergistically the knowledge gained can be applied to biodiversity management [5,6].

In the present study, we conducted the whole genome sequencing of the desert shrub *Haloxylon salicornicum* Moq. The perennial herb has a tropical distribution, however, faces the threats of extinction in the Middle-eastern region. A total data of 180 million raw sequences were generated by Illumina Hiseq 2000 sequencing that included 5,323,041,232 paired-end reads of 126 bp each with a GC content of 36.77%. The average Phred score per base was Q ≥ 40. Raw reads were *de novo* assembled into 533,304 contigs by Abyss yielding a genome of 1.5 Gb. The largest contig size and N50 was 50,005,871 bp 50,000,194, respectively (Table 1). The total number of bases in the contigs amounted to 1,550,023,735.

**Table 1:** Basic statistics and N50 and GC content of raw and assembled sequences of Haloxylon salicornicum.

*Platform*	*Illumina Hi Seq 2500*
*Total raw reads*	*180 million*
*Average read length (bp)*	*125*
*Total no. of Contigs*	*533,304*
*Max. Contig length*	*50,005,871*
*Mean Contig length*	*50,000,765.65*
*N50 value max*	*50,000,194*
*Sum of bases in contigs*	*1,550,023,735*
*GC %*	*36.77%*
*Mean Quality*	*Q ≥ 40*

**Fig. 1:**
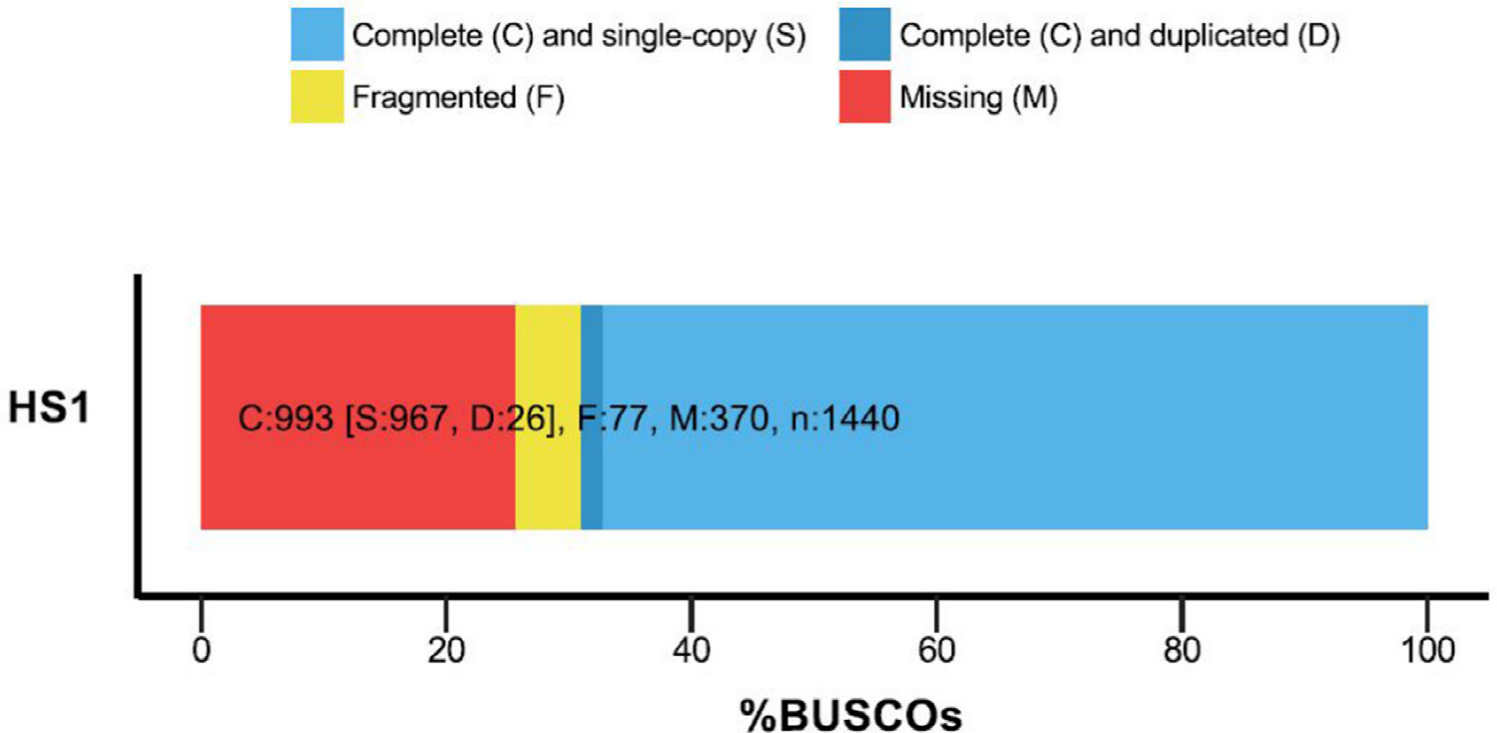
BUSCO assessment of completeness of genome.

The BUSCO evaluation of completeness of the *H. salicornicum* genome sequence predicted that it was 67% complete (Fig. 1). A total of 1,440 BUSCO groups were searched in the genome mode. The genome assembly was found to contain 967 complete single-copy BUSCOs, 26 complete duplicated BUSCOs, 77 fragmented BUSCOs, and 370 missing BUSCOs (Fig. 1).

**Fig. 2:**
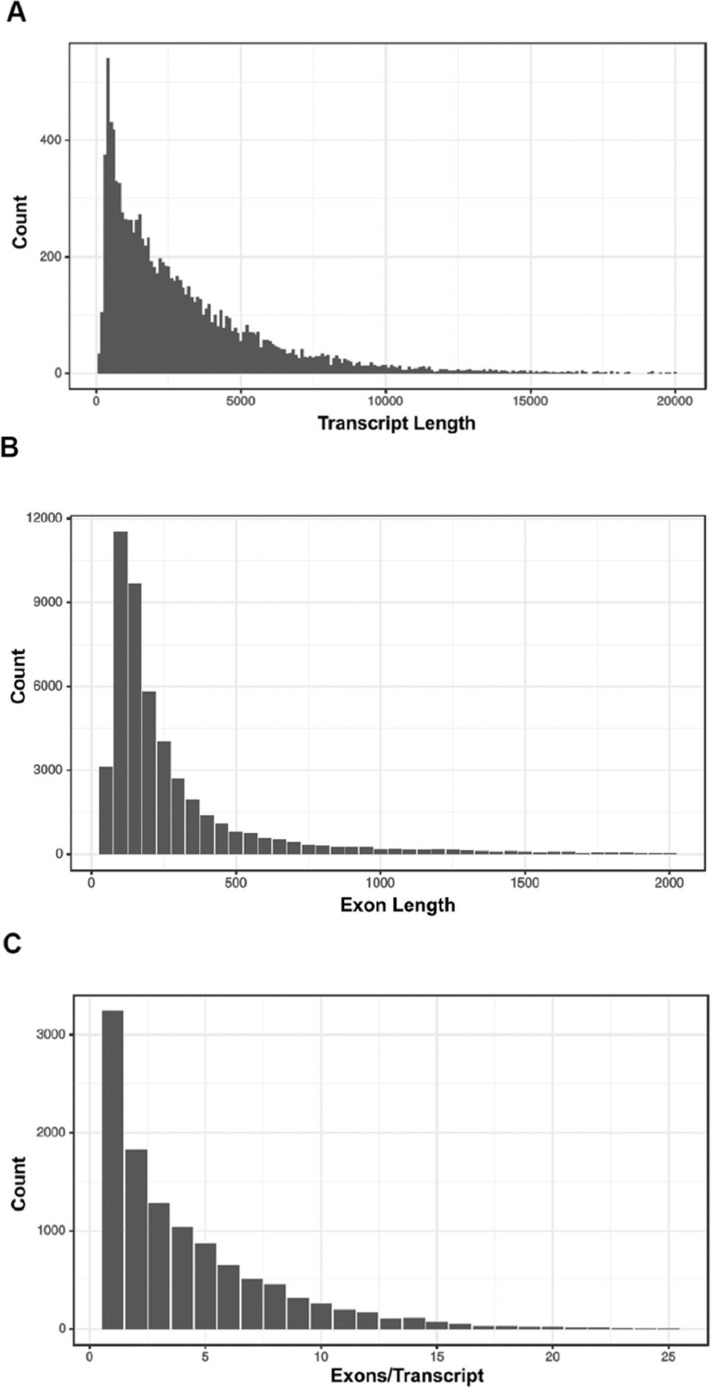
Bar plots showing (A) Distribution of transcript lengths, (B) exon lengths and (C) exons number per transcript.

Gene annotation was performed against the *H. ammondendron* transcript assembly. The repeat modeller identified 1,796,653 repeats, 28,963 est2genome, 18,682 protein2genome and 12,690 gene structures. Multiple evidences by MAKER classified the gene structures into 11,280 protein-coding genes, 11,309 mRNAs, 51,263 exons and 48,100 CDs. We compared metrics for the full set of gene models, and the smaller high confidence set for transcript lengths (Fig. 2A), exon lengths (Fig. 2B) and exons numbers per transcripts (Fig. 2C).

The average length of the predicted transcripts (mRNA) was 3,216.49 (%) with a median length of 2,287 bp. The total coding length was 13,021,732. A significant number of non-coding transcripts or introns (36,786) were also predicted with a total length of 20,829,849 as represented in Table 2. The average and median lengths of the introns were 566.24 and 216, respectively.

**Table 2:** Feature of coding and non-coding transcripts in Haloxylon salicornicum.

	Coding transcripts	Non-Coding Transcripts
*Count*	*11309*	*36786*
*Average Length*	*3216.49*	*566.24*
*Median Length*	*2287*	*216*
*Total length*	*36375287*	*20829849*

The 48,090 exons were classified based on their position and length. The exons present at the initial position were 7,582, internal was 30,050, terminal was 2,719, UTR3 were 4,124 and UTR5 were 4,105 (Table 3). The largest average size of the exon was ∼890 bp represented as single exons in the genome, whereas the minimum was ∼150 for UTR5.

**Table 3:** Exon features and their position in the annotated genome of Haloxylon salicornicum.

Exon	All	Initial	Internal	Terminal	Single	UTR3	UTR5
*Count*	*48090*	*7582*	*30050*	*7739*	*2719*	*4124*	*4105*
*Average Length*	*270.6*	*326.5*	*181.16*	*344.99*	*891.99*	*264.49*	*147.57*
*Median Length*	*149*	*198*	*123*	*225*	*660*	*239*	*106*

**Fig. 3:**
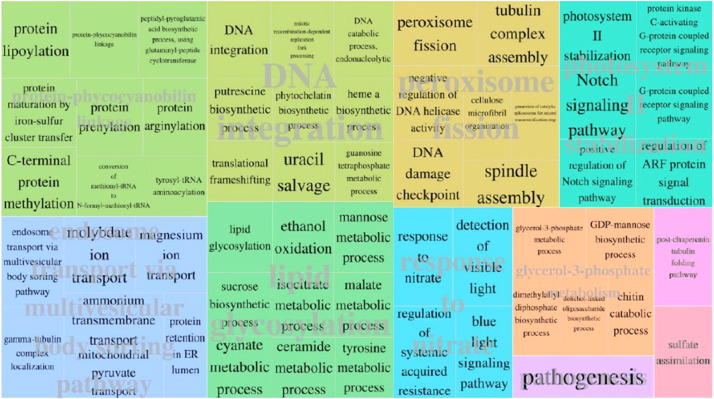
REVIGO-TreeMAP showing the distribution of gene ontology terms related to each biological process.

A total of 7,222 (64%) protein-coding sequences have been annotated out of 11,309 by at least one ontology term. All these genes were associated with 11 major biological processes branched into 60 child processes (Fig. 3).

## 2 Experimental Design, Materials and Methods

### 2.1 Preparation of plant material and DNA extraction

Fresh leaves of *H. salicornicum* were collected from a single specimen growing in the Al Kabd area of Kuwait. GPS coordinate of the collected specimen was recorded. Young leaf samples and shoots were stored in sealed polythene bags and transported on ice to the lab. The sample was appropriately labelled and kept at -80˚C until further use. DNA isolation from leaf tissues was carried out using GenElute^TM^ Plant Genomic DNA Miniprep Kit (Sigma, St. Louis, MO), as described previously [2]. The DNA isolation was done in triplicate. DNA purity (Absorbance ratio A260/A280) and quantity (Absorbance at 260 nm) were measured by the Nanodrop (Thermo Scientific, Carlsbad, CA) and Qubit fluorometer (Thermo Fisher Scientific, Carlsbad, CA). Isolated DNA samples of *H. salicornicum* were run on 0.8% of agarose gel to check the intactness and quality.

### 2.2 DNA sequencing and assembly

Genomic DNA was digested using the restriction enzymes PstI+BtgI (New England BioLabs, Inc., Ipswich, MA, United States), and barcoded adapters were ligated to each DNA sample using T4 ligase (New England BioLabs, Inc.). Dual indexed libraries were loaded across 1 lane of a 126 bp paired-end read sequencing run on the Illumina HiSeq 2000 at the University of Minnesota Genomics Centre (http://genomics.umn.edu/). The quality of the fastq files was assessed via the FastQC tool and a Q value ≥40 was recorded [7] (Fig. S1–S4). Adapters were trimmed using Trimmomatic [8]. The sample was assembled with Abyss 2.0.2 using the “abyss-pe” command setting a kmer size of 64 (k=64). Assembly statistics were generated by QUAST 3.9 [9] (Table S1). Completeness of genome was evaluated using Benchmarking Universal Single-Copy Orthologs Version 2 (BUSCO v3.0.2) [10].

### 2.3 Gene annotation

*Haloxylon ammodendron* transcriptome assembly (GSE63970_Trinity.fasta) was used to provide evidence based gene prediction in the MAKER pipeline [11]. A *de novo* gene prediction tool AGUSTUS [12] was trained using a curated dataset to use in the MAKER pipeline. A *de novo* repeat element identification was performed for repeat masking to correctly predict gene structures using Repeat Modeler [13]. Functional annotation was carried out by interproscan-5.29-68.0 [14]. They were classified into Gene ontology categories and visualized using Web Gene Ontology Annotation Plot (WEGO) 2.0 [15].

## Ethics Statement

Not applicable.

## CRediT authorship contribution statement

**Fadila Al Salameen:** Conceptualization, Writing – original draft. **Nazima Habibi:** Software, Data curation, Visualization, Writing – review & editing. **Sami Al Amad:** Funding acquisition, Supervision. **Bashayer Al Doaij:** Methodology.

## Declaration of Competing Interest

The authors declare that they have no known competing financial interests or personal relationships that could have appeared to influence the work reported in this paper.

## Appendix Supplementary materials

Image, application 1
