# Supplementary material for: Data on draft genome assembly and annotation of Haloxylon salicornicum Moq
Source: Data Brief. 2021 Dec 16;40:107721. doi: 10.1016/j.dib.2021.107721 (PMC8717446; doi:10.1016/j.dib.2021.107721)
Supplement: Supplementary file 1 [file mmc1.docx]

**Data in Brief Supplementary Data**

**QC Analysis Report**

| **Library type:** | Paired-end |
| --- | --- |
| **Fastq folder:** | /panfs/roc/umgc/illumina_analysis/170627_D00635_0260_BCB7RDANXX-analysis/demultiplex_20170706-15-27-10/demultiplex/Dweikat_Project_001 |
| **Commandline:** | /home/umii/public/gopher-pipelines/1.5/bin/umgc-singlesample.pl –gz –projectfolder /panfs/roc/umgc/illumina_analysis/170627_D00635_0260_BCB7RDANXX-analysis/demultiplex_20170706-15-27-10/demultiplex/Dweikat_Project_001 –name 170627_D00635_0260_BCB7RDANXX –threads 20 –scratchfolder /panfs/roc/scratch/umgc_bin/170627_D00635_0260_BCB7RDANXX/demultiplex_20170706-15-27-10/Dweikat_Project_001/illumina-basicQC –outputfolder /panfs/roc/umgc/illumina_analysis/170627_D00635_0260_BCB7RDANXX-analysis/demultiplex_20170706-15-27-10/demultiplex/Dweikat_Project_001/Analysis/illumina-basicQC/illumina-basicQC |

**Fastq Quality Plots**


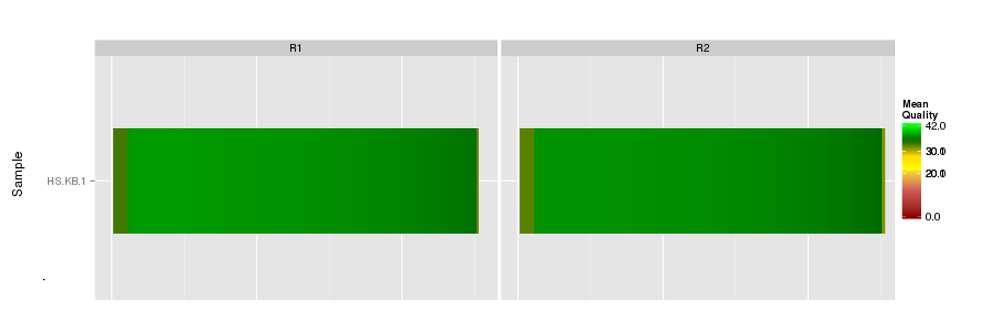


**Fig S1. Quality distribution of raw reads of *Haloxylon salicornicum***

**Fastq Screen Plots**


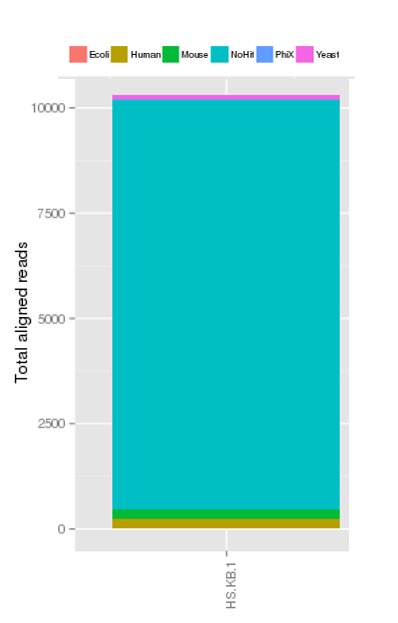


**Fig S2. Data Alignment**

**Summary Plots**

**
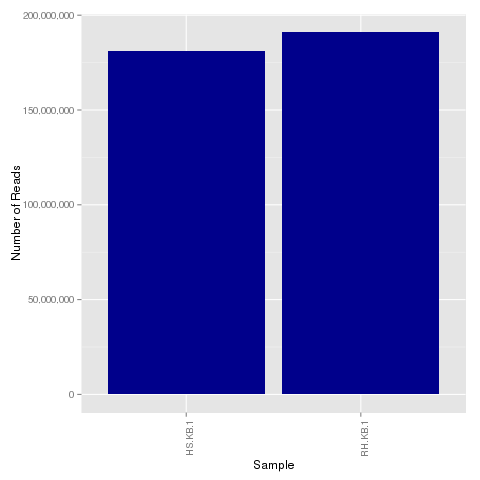
**

**Fig S3. Raw reads generated by paired end sequencing**

**Percent GC Content**


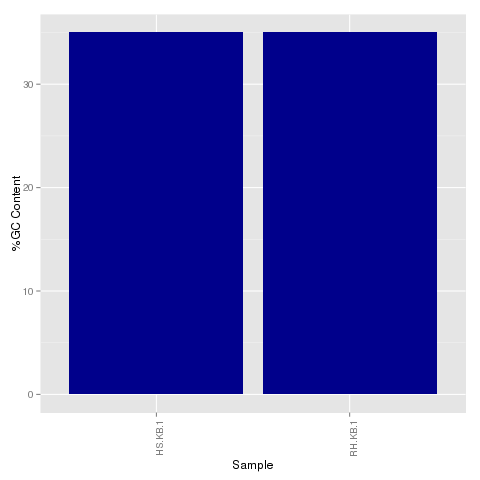


**Fig S4. Percent GC content of *Haloxylon salicornicum***

**QUAST Assembly statistics**

**Table S1 Basic statistics of the assembled genome of *H. salicornicum***

| # contigs | 31 |
| --- | --- |
| # contigs (>= 0 bp) | 31 |
| # contigs (>= 1000 bp) | 31 |
| Largest contig | 50005871 |
| Total length | 1550023735 |
| Total length (>= 0 bp) | 1550023735 |
| Total length (>= 1000 bp) | 1550023735 |
| N50 | 50000194 |
| N75 | 50000071 |
| L50 | 16 |
| L75 | 24 |
| GC (%) | 36.77 |
